# Orchestrating the Matrix: The Role of Glial Cells and Systemic Signals in Perineuronal Net Dynamics

**DOI:** 10.1007/s11064-025-04506-8

**Published:** 2025-07-29

**Authors:** Valentino Totaro, Tommaso Pizzorusso, Paola Tognini

**Affiliations:** 1 BIO@SNS Lab, Scuola Normale Superiore, Pisa, Italy; 2 Institute of Neuroscience, National Research Council, Pisa, Italy; 3 Health Science Interdisciplinary Center, Scuola Superiore Sant’Anna, Pisa, Italy

**Keywords:** Perineuronal nets, Extracellular matrix, Microglia, Astrocytes, Glycosaminoglycan, Plasticity, Metabolism

## Abstract

Perineuronal nets (PNNs) are specialized, dense extracellular matrix structures that enmesh the cell bodies and dendrites of specific neurons, most notably inhibitory interneurons. Increasing evidence indicates that PNNs serve not merely as passive scaffolds but play an active and essential role in modulating synaptic plasticity and circuit physiology. They critically influence the timing of sensory system critical periods, as well as processes underlying learning, memory, and higher cognitive functions. Furthermore, dysregulation of PNN density and architecture have been associated with conditions like autism, neurodevelopmental disorders, schizophrenia and Alzheimer’s disease. Since they are extensively involved in brain function, we discuss the multitude of regulatory factors that govern the formation, maturation, and remodeling of PNNs. In particular, we focus on both molecular and cellular brain-intrinsic mechanisms, highlighting the potential contributions of microglia and astrocyte derived factors. Additionally, we consider the influence of long-range signaling cues, including the metabolic status and peripheral hormones. Analysing this complex network of interactors, we try to highlight the role of PNNs beyond neural plasticity and brain function, in a broader whole-body physiological perspective.

## Introduction

Neural plasticity, the ability of the brain to adapt and reorganize, is essential for learning, memory, and recovery from injury. Perineuronal nets (PNNs), dense extracellular matrix (ECM) structures enwrapping the soma and dendrites of a large number of neurons in the brain, represent crucial regulators of such processes.

**Fig. 1:**
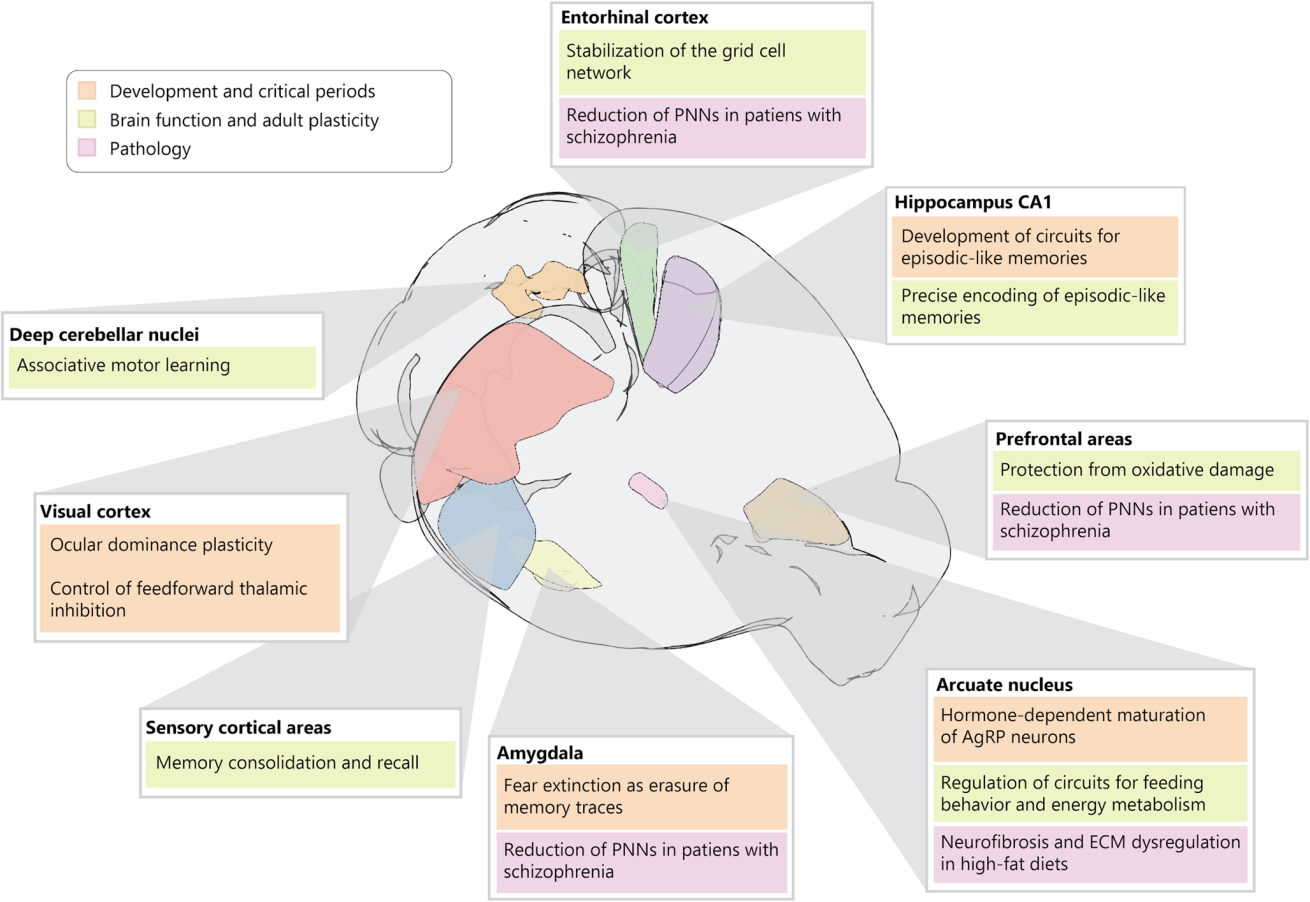
Functional roles of PNNs across multiple brain regions. Regional specificity of PNN-mediated control of neural plasticity is suggestive of the action of complex regulatory networks both in postnatal development and in adulthood. The cartoon of the mouse brain was created with Brainrender [43] and adapted for illustration purposes

Interest in PNNs has steadily increased over the past two decades, as their involvement in key brain functions has been demonstrated across multiple brain regions (Fig. 1). By antagonizing plasticity, PNNs promote the maturation of neural circuits during early postnatal life, gating the temporal windows of maximal development called *critical periods*. This role was first established in ocular dominance plasticity within the visual cortex [1], and later extended to various functions, including fear extinction in the amygdala [2], AgRP neurons maturation in the arcuate nucleus of the hypothalamus [3, 4], precise encoding of episodic-like memory in the hippocampus CA1 [5, 6].

During adulthood, PNNs stabilize acquired neural representations and memories in multiple regions. PNN depletion affects spatial maps in the grid cell network [7], fear memory acquisition, consolidation and extinction [8–10], cerebellar plasticity and related learning [11].

From a molecular point of view, PNNs are composed of a lattice-like scaffold of hyaluronic acid chains and chondroitin-sulfate proteoglycans (CSPGs), connected by link proteins and tenascin-R [12, 13]. While sharing some molecular constituents with the unorganized ECM of the brain, PNN-bound CSPGs display specific sulfation motives and can be isolated through biochemical methods [14]. In histological preparations, many PNNs are labeled by the lectin *Wisteria floribunda agglutinin* [15], which is one of the most common reagents used as PNN marker. Despite the common structural organization, PNNs can display variable proteoglycan composition, such that some PNNs can be detected by specific antibodies but not by WFA [16–18].

Due to their tight involvement in plasticity processes, PNNs are associated with various pathological conditions. In the context of brain injury, CSPG expression is upregulated in the glial scar and, in the dorsal root ganglia, acts as an inhibitor of axon growth [19–21]. Disassembly of PNNs by the bacterial enzyme chondroitinase ABC (ChABC) can promote recovery after spinal cord injury [22, 23]. In other systems, the inhibition of axon growth has been shown to limit plasticity and has been attributed to the action of the chemorepulsive molecule semaphorin 3A (SEMA3A), which can bind the PNN scaffold [24–26].

While the limitation of neuroplasticity is sometimes viewed as a constraint, it also serves essential functions. Controlled plasticity ensures stability in neural circuits, and prevents maladaptive changes. Alterations in PNNs have indeed been reported in multiple models of neurodevelopmental disorders [27–31], in which impaired plasticity severely affects cognitive, motor and behavioral function.

In addition to their plasticity-limiting role, PNNs and their molecular constituents can display a neuroprotective function [32]. The reduction of PNNs around parvalbumin-expressing (PV) interneurons has been related to an increase in oxidative stress in animal models and in post-mortem samples of patients with schizophrenia [33–35]. A reduction of WFA signal has also been reported in neurodegenerative pathologies such as Alzheimer’s disease [36]. However this notion has been challenged [37] and the reduction in the staining imputed to a change in PNN composition or sulfation pattern altering WFA reactivity [27].

Despite the general role in controlling plasticity in multiple brain areas, PNN distribution in the adult brain is not uniform. In the mouse, the highest levels are found in the midbrain, the hindbrain and the cortex, particularly in primary sensory regions [38]. PNNs are typically associated with PV inhibitory neurons, although they can also encircle different cell types, for instance pyramidal neurons in the hippocampus CA2 [39].

The regional heterogeneity in PNN distribution and composition, as well as in the time course required for their formation suggest that these structures are tightly regulated and controlled across the entire lifespan. The mechanisms underlying PNN formation and maturation are crucial for circuit-specific control of plasticity.

While the functions of PNNs in physiological and pathological contexts have been extensively reviewed [12, 40–42], here we explore the complex network of regulatory elements that govern the formation, maturation, and remodeling of PNNs across development and into adulthood. We place particular emphasis on both molecular and cellular factors within the CNS and extrinsic signals originating from peripheral tissues. Besides purely neuronal signals, we will also consider regulation by glial cells, specifically astrocytes and microglia. These cell types are now intensely studied for their capability to act locally while still sensing and integrating systemic factors. Although much attention has traditionally been given to brain-intrinsic mechanisms, emerging evidence suggests that systemic cues, such as metabolic signals, hormones, and inflammatory mediators, can also influence PNN dynamics and architecture. By examining how central and peripheral signals converge to regulate PNN formation, we aim to shed light on the multifaceted role of these ECM structures beyond neural plasticity and brain function, in broader physiological processes such as energy homeostasis.

## Molecular and Physiological Factors Modulate PNN Aggregation during Development

**Fig. 2:**
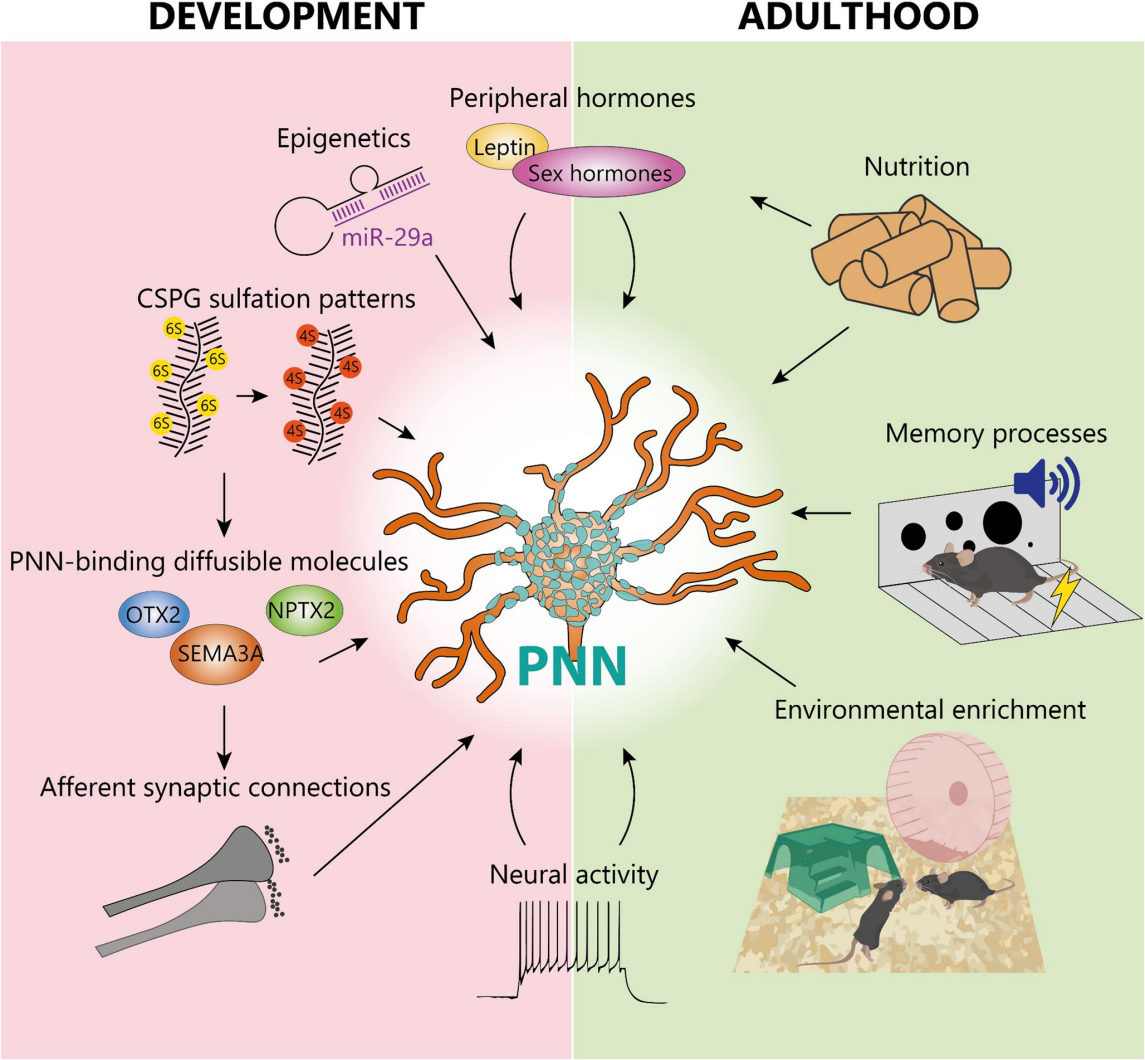
Regulation of PNNs during development and adulthood. Molecular and physiological factors promoting circuit maturation during postnatal development are involved in the accumulation of PNNs. During adulthood, events requiring plasticity, like memory formation and consolidation, affect PNN maintenance and function. Notably, PNNs integrate both brain-intrinsic and systemic cues, such as nutrition and hormonal landscape

Regulating neural plasticity during early postnatal life is essential to guide neural circuits along a healthy developmental path, ultimately enhancing their functional efficiency in adulthood. For these reasons, the formation of PNNs is not a completely spontaneous event but instead an actively regulated process that must be initiated and timely controlled (Fig. 2).

PNNs are highly complex molecular structures, composed of a variety of proteins and CSPGs. Therefore, the biosynthetic processes of each of these constituents offer multiple regulatory hubs that enable tight control over PNN formation.

The building blocks of PNNs are produced by different cell types, including neurons, astrocytes and oligodendrocytes [40]. Although many CSPGs are already present during embryonic development and serve as extracellular cues to orchestrate neurogenesis, cell migration, and axon guidance, the expression of aggrecan, brevican, and link proteins increase postnatally during PNN formation [44, 45]. These components are essential for the physiological formation of PNNs. Indeed, their removal has been shown to result in attenuated PNNs and persistent plasticity. This phenotype is observed following the knock-out of hyaluronan and proteoglycan link protein 1 (Hapln1) [45], the combined removal of multiple ECM proteins like tenascins and CSPGs [46], and the genetic deletion of aggrecan in adults [47]. In contrast, removing aggrecan from birth (germline knock-out) surprisingly had no effect on plasticity or PV neuron physiology, suggesting that the developing brain activates compensatory mechanisms to offset its loss [48]. In several systems, the expression PNN molecules is linked to neural activity during early postnatal life, which promotes circuit maturation. For instance, sensory deprivation delays critical period closure and PNN formation in the visual [1] and somatosensory [49] cortex, with concurrent reduction of the expression of aggrecan.

In addition to the production of molecular components, studies have shown the importance of sugar moiety modifications, particularly the sulfation patterns of glycosaminoglycans, as a regulatory ‘code’ that modulates PNN function. As mentioned before, CSPGs are crucial elements of PNN scaffold and are composed of a core protein to which a number of chondroitin sulfate glycosaminoglycan (CS-GAG) chains are attached. During the synthesis of CS-GAG chains, sulfate groups are added by sulfotransferase enzymes [50] at carbon 4 and/or 6 of N-acetylgalactosamine. The pattern of sulfation results in charged domains that affect the binding properties of CSPGs and influence their permissiveness towards plasticity. Particularly, chondroitin-4-sulphate (C4S) has been shown to be inhibitory towards axonal growth and plasticity while chondroitin-6-sulphate (C6S) is more permissive [51]. Consistently, CSPG sulfation changes across development and lifespan: C6S is much more abundant than C4S during embryogenesis and decreases during postnatal development, while C4S increases. Their ratio stabilizes up until the onset of critical periods, after which C4S becomes predominant in the adult brain [14, 52, 53].

Due to their proximity to the cell membrane and to the biochemical properties of CSPGs, PNNs can capture and concentrate diffusible molecules acting as long-range signaling cues. Long-range signaling is crucial for coordinating the maturation of functionally related networks. Epidermal growth factor (EGF) has been shown to suppress development of GABAergic neurons in the rodent cortex. Administration of EGF to cultured neurons and EGF overexpression in transgenic mouse models reduces aggregation of PNNs around PV neurons, increasing the enzymatic activity of matrix metalloproteinases (MMPs) and A disintegrin and metalloproteinase with thrombospondin motifs (ADAMTS) [54]. Signaling molecules can also integrate in the PNN scaffold and regulate developmental plasticity. One such molecule, SEMA3A, acts as an axonal chemorepellant that accumulates in PNNs during late postnatal development. This accumulation restricts the formation of new synapses on PNN-enwrapped neurons [24], thus contributing to the end of the critical period [26]. Another critical protein is orthodenticle homeobox protein 2 (OTX2), which is essential for PNN development and maintenance. The understanding of OTX2 origin has evolved; it was first identified as being transported from the retina to the primary visual cortex in an activity-dependent manner [55]. However, subsequent work revealed that OTX2 is also synthesized and secreted globally by the choroid plexus, a source whose disruption affects remote circuits like the primary visual cortex [56]. While OTX2 and SEMA3A are thought to bind 4,6 di-sulfated GAGs in the PNNs [57, 58], neuronal pentraxin 2 (NPTX2) binds to both 4,6 sulfated GAGs and HA and promotes maturation of PNNs in cell cultures [59].

The action of PNNs in critical period timing is also regulated by epigenetic mechanisms. For instance, miR-29a is a microRNA upregulated with age across different species and tissues [60] and its upregulation during PNN formation can lead to the early appearance of PNNs. Conversely, inhibiting miR-29a in adult mice promoted plasticity, reduced PNNs, and increased the expression of ECM remodeling enzymes such as MMP2, MMP9, MMP13 [61].

In some brain regions, PNNs are also associated with specific connectivity patterns. In the cortex, the highest levels of PNN expression is found in the thalamic recipient layer 4 of primary sensory cortices and in PV-rich areas connected into intracortical networks [38]. However, while it has been shown that in the primary visual cortex PNN degradation can induce an activity-dependent increase in thalamic afferents onto PV neurons [62], it is not known whether those synapses might be important for instructing or facilitating PNN development. In general, further studies will be needed to elucidate the influence of connectivity in shaping PNN function.

These regulatory elements do not act independently, but actually cooperate for proper critical period timing and circuit maturation. The changing sulfation pattern of PNNs alters significantly the interactions with signaling molecules. Mice overexpressing chondroitin-6-sulfotransferase, the enzyme responsible for the juvenile C6S sulfation, show an abnormal development of PNNs in the visual cortex, revealed by reduced reactivity to WFA, and lower accumulation of OTX2 both before and after the critical period closure. This ultimately results in impaired formation of thalamocortical synapses onto PNN + neurons and retention of ocular dominance plasticity in adulthood [63].

Together, these findings underscore that PNN formation is a tightly orchestrated process, and that these structures integrate multiple intrinsic molecular cues, activity-dependent mechanisms, and biochemical signals to ensure proper maturation of neural circuits.

## Experience-dependent Regulation of Perineuronal Nets in Adulthood

While neural plasticity is most prominent during development, it remains essential in adulthood for processes such as learning, memory, and adaptive responses to experience. Therefore, multiple factors continue to regulate PNNs in the adult brain to balance stability with the need for ongoing plasticity, ensuring neural circuits remain adaptable while preserving functional integrity (Fig. 2).

As previously mentioned, the process of memory formation, consolidation and maintenance require intact PNNs, as they are impaired by PNN depletion obtained with local injection of PNN-degrading enzyme ChABC [2, 8, 11]. Consequently, experience can shape the PNN to support stable memory encoding. Nevertheless, the molecular mechanisms coupling learning to PNN expression have not been completely characterized. Memory acquisition and consolidation typically induce an increase in the expression of PNNs and of its molecular components. Auditory fear memory formation, for instance, is accompanied by an upregulation of the mRNA expression of PNN proteoglycan components like aggrecan, brevican and neurocan in the auditory cortex associated with increased numbers of aggregated PNNs [9]. PNNs around PV neurons increase 4 and 96 h after contextual fear conditioning in the hippocampal CA1 and in the anterior cingulate cortex, enhancing consolidation and recall of both recent and remote fear memory [64].

During learning, specific signaling pathways can act on plasticity to interfere with PNN expression. Neuropeptide Y (NPY) signaling has been associated with learning and memory [65]. Mice lacking NPY receptor in excitatory forebrain neurons display slower spatial learning and increased intensity of PNN in the hippocampus CA1. Deletion of PNNs with ChABC rescues the learning deficit [66].

As in development, another factor potentially capable of reshaping PNNs, promoting plasticity phenomena and paving the way for circuit rearrangements is neural activity. Indeed, in the cortex, chemogenetic inhibition of either excitatory or PV-expressing inhibitory neurons triggers a decrease in PNN density, highlighting the dependence of these structures on patterns of activity of the local microcircuit [67].

While the dependence of PNNs on neural activity has been consistently shown in multiple studies, the mechanism underlied by this regulation is not completely understood. A possible way by which PNNs can respond to neural activity is the controlled expression of activity-dependent genes that modulate the ECM. NPTX2, for instance, is a member of the neuronal pentraxin family of calcium-dependent lectins and is regulated as an immediate-early gene [68]. By binding both PNNs and AMPA receptors, it facilitates receptor clustering and excitatory neurotransmission onto PV cells [69]. In the mature brain, PNNs are engaged in a positive feedback loop mediated by PNN-dependent constant internalization of OTX2 into PV neurons, which in turn dampens adult plasticity and promotes PNN maintenance [57].

Environmental factors and experience can reactivate plasticity in the adult brain. For example, rearing mice in an enriched environment (EE)—a paradigm that enhances sensory, cognitive, and social stimulation—can promote reactivation of juvenile plasticity and, consistently, a reduction of PNNs in the visual cortex [70]. Similarly, in the cerebellum, EE is associated with a reduction of SEMA3A bound to the PNN scaffold, consistent with an increased plasticity [25]. Conversely, environmental enrichment during early postnatal life accelerates cortical development and PNN accumulation by overexpression of insulin-like growth factor 1 (IGF1) [71]. Changes of the same sign can be found in the striatum, where EE causes an increase of PNN expression [72].

Altogether, these findings demonstrate that PNNs in the adult brain are dynamic structures, shaped by learning, neural activity, and environmental experience. This regulation maintains a balance between stability and plasticity, supporting both memory consolidation and adaptive circuit remodeling. Elucidating the molecular and activity-dependent mechanisms underlying PNN dynamics will be key to understanding how experience influences long-term cognitive function and to developing strategies for modulating plasticity in neurological disorders.

## The Perineuronal Nets are Dynamic Players at the Interface between Metabolism and Brain Plasticity

As described in the previous sections, PNNs were initially characterized in cortical areas and were associated with sensory processing and memory [12]. Recent evidence highlights the presence and critical importance of PNNs in brain regions governing metabolic homeostasis, including the hypothalamus. Furthermore, their regulation appears far more complex than previously thought, extending beyond local neuronal activity to involve long-range signals related to sex, hormones, diet, and overall metabolic status.

PNN-like structures have been identified within the arcuate nucleus of the hypothalamus (ARC) [3], a critical hub for integrating peripheral metabolic signals to control energy balance and glucose homeostasis [73]. These ARC PNNs are unique in several ways. They form a dense, continuous ring or ‘collar’ at the junction between the ARC and the median eminence (ME), a circumventricular organ providing neurons direct access to circulating hormones and nutrients since the blood brain barrier (BBB) composed of fenestrated microvessel loops is particularly permeable [74]. This dense clustering contrasts sharply with the sparser distribution of PNNs seen in cortical regions. Notably, ARC PNNs seem to preferentially enmesh metabolically relevant neuronal populations. The majority are GABAergic, leptin-receptor positive (LepRb+), and include most Agouti-related peptide (AgRP) neurons—key orexigenic regulators—while only a smaller fraction of anorexigenic pro-opiomelanocortin (POMC) neurons are PNN-enmeshed. Postnatal appearance of these PNN, becoming fully formed around postnatal day 28 (P28), coincides precisely with the closure of the critical period for the maturation of AgRP neuron projections and the establishment of their response to leptin [3]. Thus, the timing of ARC PNN formation is tightly linked to postnatal neurodevelopment and, likely, to hypothalamic plasticity, in line with PNN maturation in other brain regions [1, 75]. Compelling evidence indicates that this developmental process is dependent on long-range hormonal signals, specifically the adipocyte-derived hormone leptin. In leptin-deficient *ob/ob* mice, ARC PNN formation during the postnatal period is markedly impaired. This deficit can be rescued by systemic leptin administration, however only if given during the critical period [3]. This establishes leptin signalling as a key factor not only for AgRP neuron development [76] but also for the assembly of the surrounding PNNs. This piece of data strongly implicates PNNs in the mechanisms that close the critical developmental window for metabolic circuit wiring and suggests the PNN as potential stabilizer of hypothalamic feeding circuits.

Beyond development, hypothalamic PNNs exhibit ongoing remodelling and regulation in adulthood, influenced by sex, gonadal hormones, and metabolic status. Studies in adult mice revealed that gonadal hormones are essential for maintaining the structural integrity of PNNs specifically within the ARC. a portion of PNN-enmeshed ARC neurons express estrogen receptor α (ERα) and a majority express the androgen receptor. Indeed, surgical gonadectomy significantly reduced both WFA intensity and the number of PNN-enwrapped cells in the ARC, an effect observed in both sexes. Intriguingly, the terete hypothalamic nucleus (TE) shows significant sex dimorphism, with females displaying higher PNN WFA fluorescence intensity than males. Furthermore, PNNs in the TE are sensitive to diet in a sex-specific manner; chronic high-fat diet (HFD, 60% Kcal from fat) feeding significantly increased PNN intensity in females but had no effect in males. Finally, PNNs observed in other hypothalamic nuclei, such as the paraventricular hypothalamic nucleus(PVH), lateral hypothalamic area (LHA), ventromedial hypothalamic nucleus (VMH), and dorsomedial hypothalamic nucleus (DMH), did not show significant regulation by sex or HFD consumption [77].

The ME is another key site of PNN plasticity directly linked to the nutritional state. PNNs in the ME undergo rapid remodeling in response to acute changes in the feeding state. Kohnke et al. demonstrated that refeeding after an overnight fast triggers a rapid increase in the proliferation and differentiation of oligodendrocyte progenitor cells (OPCs) into mature oligodendrocytes specifically within the ME. Intriguingly, this nutritionally-driven oligodendrocyte differentiation is directly linked to PNN remodeling. Fasting reduces, while refeeding rapidly increases, the density of ME PNNs. This reshaping is dependent on OPC differentiation, as the deletion of the transcription factor Myelin regulatory factor (Myrf) in OPC blunts the PNN response to refeeding. Furthermore, the PNN-degrading enzyme ADAMTS4 shows increased expression in ME oligodendrocytes during fasting, potentially contributing to PNN breakdown. Importantly, this rapid PNN remodeling in the ME is functionally relevant for energy balance. Enzymatic digestion of ARC/ME PNNs using ChABC in lean mice led to increased food intake and weight gain, demonstrating that intact ME PNNs are required for normal energy homeostasis maintenance [78]. This results highlights the ME as a site of unique glial and ECM plasticity directly responsive to acute nutritional challenges, influencing central metabolic control and mouse feeding responses.

The remodelling of PNNs in key hypothalamic nuclei for metabolic homeostasis maintenance may be crucial for the development and progression of pathological states. Recent evidence indicates that established metabolic diseases (obesity and type 2 diabetes) are associated with a distinct, pathological PNN alteration in the ARC, termed neurofibrosis. In both high fat high sugar (HFHS, 45% kcal from fat) diet-induced obese (DIO) and genetically diabetic (db/db) mice, the ARC shows significant remodeling of the PNN, including increased WFA staining, elevated aggrecan deposition, and altered CS-GAG sulfation (notably a higher 4 S/6S ratio). These changes occur specifically around AgRP neurons and progress with the metabolic disease. The authors hypothesized that neurofibrosis is the result of reduced PNN turnover in obesity. In DIO mice, PNN degradation is slower than in lean controls and is associated with lower expression of ECM proteases. Functionally, ARC neurofibrosis impairs insulin access to neurons by forming a physical and electrostatic barrier. Hypothalamic insulin signaling, tested through phosphorylation of the insulin receptor and AKT, is reduced in DIO mice but restored by enzymatic PNN removal with ChABC, which mechanistically should disrupt the insulin-GAG binding and sequestration. Remarkably, stereotaxic injections of ChABC in DIO or *db/db* mouse models led to metabolic improvements. To increase translatability, CS-GAG synthesis inhibitor fluorosamine intranasal administration to DIO mice decreased body weight, adiposity, restored glucose tolerance and insulin sensitivity. Together, these findings suggest ARC neurofibrosis is a causal factor in neuronal insulin resistance and dysfunction in obesity, and a novel potential druggable target [79].

In contrast to this discovery, a different study demonstrated that the abundance of PNNs enmeshing ARC neurons is decreased in DIO mice compared to lean control diet fed animals. This loss was also associated with increases of ARC microgliosis and astrogliosis suggesting a link between ARC PNN loss and glial activation. ChABC-driven depletion of ARC PNNs exacerbates the metabolic impact of a HFHS diet in rats. Specifically, PNN digestion led to increased hyperphagia, greater weight gain, and elevated fat accumulation, primarily driven by enhanced food intake [80]. On the same page, compared to healthy Wistar controls, Zucker diabetic fatty (ZDF) rats, a model of type 2 diabetes, exhibit markedly reduced PNN abundance in the ARC and altered sulfation pattern of their CSPG. A single intracerebroventricular injection of fibroblast growth factor 1 (FGF1) induces sustained remission of diabetes in ZDF rats and increases ARC PNN density, normalizing toward healthy levels. Remarkably, PNN digestion through ChABC administration completely prevents diabetes remission, indicating that the integrity of ARC PNNs is essential for the sustained glucoregulatory effect of FGF1 [4].

These contrasting findings suggest that ARC PNNs may play a role in energy balance, and that their dysregulation could contribute to the development of obesity and insulin resistance. However, further investigation is needed to determine whether they are causative, and how they might be effectively targeted.

The influence of diet, particularly HFD, on PNN plasticity extends beyond the hypothalamus. Studies in the prefrontal cortex (PFC) reveal complex, region-specific, and sex-dependent effects of HFD on PNNs, which also vary based on inherent susceptibility to obesity. In obesity-prone (OP) and standard Sprague-Dawley (SD) male rats, chronic HFD reduced PNN intensity in the prelimbic (PL) and ventromedial orbitofrontal (vmOFC) subregions. In contrast, HFD increased PNN intensity in the infralimbic (IL) PFC of female OP/SD rats. Obesity-resistant (OR) rats showed opposite responses, with males exhibiting increased PNN intensity in the vmOFC and females showing decreased intensity in the IL-PFC after HFD [81]. Given that PFC PNNs typically surround PV + interneurons and regulate E/I balance, these diet- and sex-specific changes likely impact PFC function related to cognitive control, decision-making, and motivation, potentially contributing to difficulties in voluntary food consumption. HFD has been associated with excessive accumulation of PV-positive neurons and upregulation of PNN in the mouse CA3 region of the hippocampus. Treatment with indoleamine 2,3-dioxygenase (IDO) inhibitor 1-methyltryptophan (1-MT) improved performance in the tails suspension test and forced swimming test, and effectively reverses ECM changes, highlighting a PNN-related mechanism by which 1-MT exerts its antidepressant effect [82]. HFD consumption decreases the number of WFA + cells in the CA1 hippocampal region of WT mice; and a mouse model knock-out for pleiotrophin, a cytokine implicated in HFD-induced weight gain, brain alterations and cognitive impairments, displays a higher number of WFA + cells, which are not affected by HFD [83].

The studies reviewed here powerfully illustrate that PNN formation, maintenance, and remodeling are not solely governed by local neuronal activity or developmental programs within the brain. Instead, PNNs in metabolic circuits exist at a nexus, integrating both local and long-range signals. Peripheral metabolic/sexual hormones regulate PNN formation, and intriguingly the systemic metabolic status influences PNN assembly and turnover in the hypothalamus. This complex interplay underscores the role of PNNs as dynamic integrators, situated at key brain–body interfaces like the ARC-ME site, translating peripheral metabolic information into structural changes that regulate neuronal function and likely circuit stability, impacting whole-body energy homeostasis.

## Astrocytes Regulate ECM in Health and Disease

**Fig. 3:**
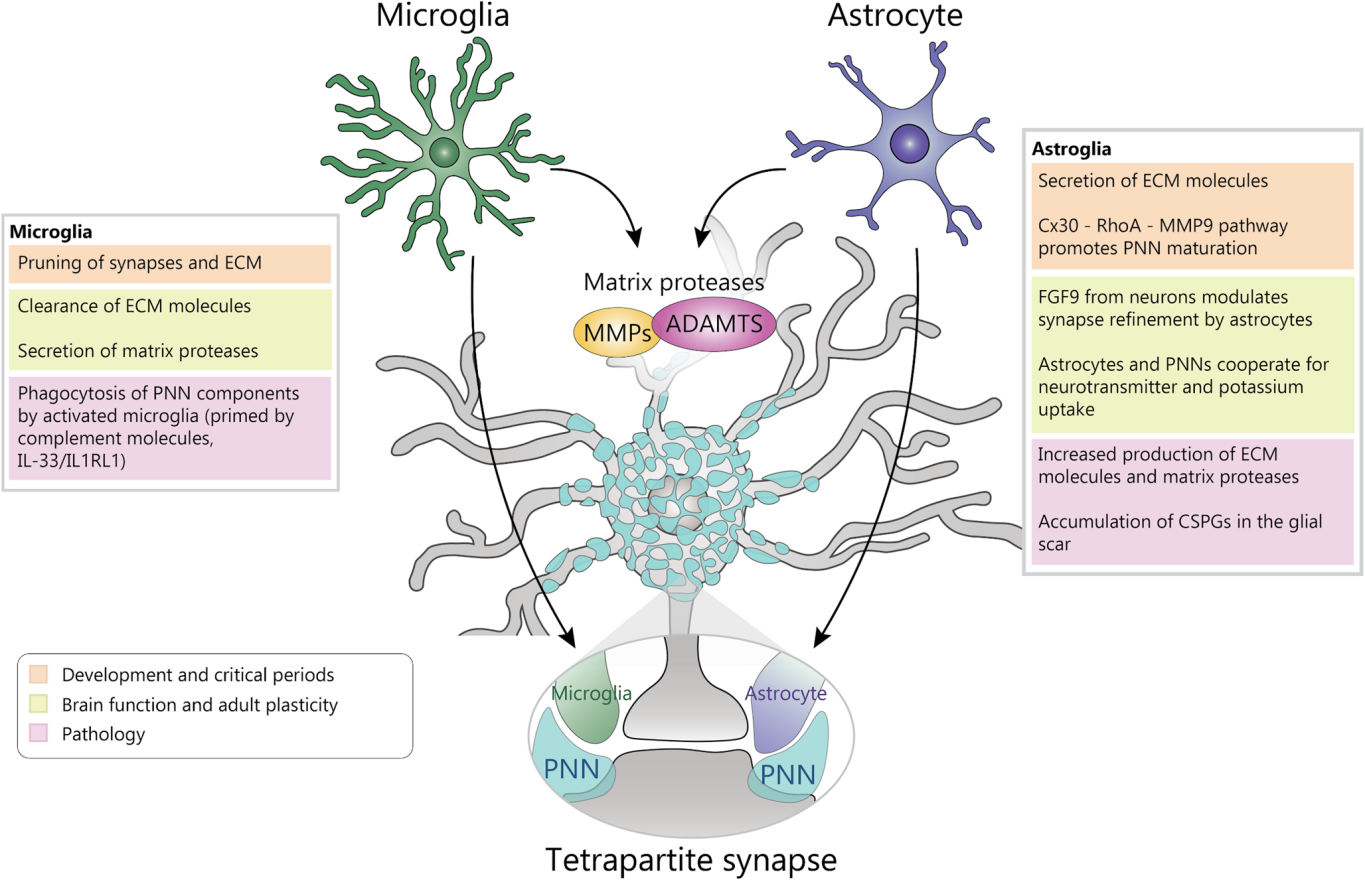
Regulation of PNNs by glial cells. Microglia and astrocytes influence PNN development and stability both in physiological and in pathological conditions. Microglia is mostly associated with clearance of ECM molecules while astrocytes with their production. Both cell types are involved in regulation of PNNs by matrix metalloproteases and in synaptic homeostasis

Glial cells, historically viewed as passive scaffolding, are now understood to play dynamic and regulatory roles in neural signaling and plasticity. Astrocytes, the most abundant glial cell type in the brain, are crucially involved in the maintenance of potassium homeostasis and in the uptake of glutamate at synapses [84]. Moreover, growing evidence increasingly recognized them as active participants of the development, refinement and maintenance of neural circuits through different processes including a direct influence on PNN expression [85, 86] (Fig. 3).

Astrocytes are a substantial source of ECM components throughout development and adulthood. However, the fraction of these molecules eventually incorporated in the developing PNNs is still debated. PNN-like structures have been reported in cultures of neurons without astrocytes [87], suggesting that neuron-secreted molecules might be sufficient for the assembly of PNNs. Consistently, co-cultures experiments of wild-type astrocytes and neurons lacking expression of tenascin-R, tenascin-C, neurocan and brevican showed lower expression of aggregated PNNs [88]. In vivo knock-out of these molecules, however, had resulted in limited impairment of PNN assembly, possibly due to compensatory mechanisms [89, 90].

During the visual cortex critical period, astrocytes increase their expression of the gap junction channel subunit connexin 30 (Cx30). Through an atypical signaling pathway, Cx30 downregulates the RhoA-matrix metalloproteinase 9 (MMP9) pathway. This cascade leads to reduced levels of MMP9, an enzyme that degrades ECM components, consequently promoting the maturation and condensation of PNNs around inhibitory neurons and contributing to the closure of critical period for ocular dominance [86]. In vivo transplantation of mature astrocytes in the developing cortex results in anticipated gating of developmental plasticity [86]. An astrocyte-driven control of PNNs can be found also in subcortical regions which are typically less studied for PNN biology. In the medial nucleus of the trapezoid body (MNTB), PNNs ensheat principal neurons and the large calyx of Held terminals [91]. Disruption of fibroblast growth factor 9 signaling between neurons and astrocytes in MNTB results in higher brevican (BCAN) accumulation inside PNNs and concurrent increased size of calyx of Held terminals, indicating a role of this communication in synapse refinement [92].

In the adult brain, astrocytes are essential for synaptic homeostasis as they intimately interact with both the pre- and post- synaptic terminals, in tight association with the ECM and microglia creating structures increasingly termed ‘tripartite’ or even ‘tetrapartite’ synapses ([93], Fig. 3). The association is particularly strong in the meshes of the PNN lattice: here most afferent terminals making contacts with the postsynaptic membrane are accompanied by astrocytic processes expressing Kir4.1, glutamate and GABA transporters [94]. The degradation of PNNs impairs astrocytic transmitter and potassium uptake with consequent spillage of glutamate in the extrasynaptic space, indicating that PNNs are necessary for physiological regulation of synaptic physiology by astroglia [94].

The interplay between ECM and astrocytes is also evident in pathological conditions. In disease and neuroinflammatory conditions astrocytes transition in a so-called reactive state undergoing morphological and molecular changes [95, 96], such as the increased expression of glial fibrillary acid protein (GFAP), an intermediate filament protein, which is widely used as an astrocytic marker [97]. Reactive astrocytes also alter ECM homeostasis by increasing the expression of ECM components and matrix metalloproteases, increasing the levels of interstitial ECM while reducing aggregated PNNs [98]. In pathologies involving brain injury, reactive astrocytes take part in the formation of the glial scar [99], accumulating various CSPGs that counteract axonal regrowth [100, 101]. On the other hand, upon injury astrocytes also increase MMP2, MMP3 [102, 103]. Upon disruption of the blood brain barrier and albumin extravasation, TGFβ signaling in reactive astrocytes promote PNN degradation [104].

It is worth noting that it is still hard to disentangle the action of astrocytes on aggregated PNNs from their regulation of the diffuse ECM. Astrocytes exert a broad action on several ECM components and the regulation of PNNs can be considered part of a much broader picture. Recent evidence showed that during synaptogenesis astrocytes secrete neurocan (NCAN) that is cleaved in two fragments. The one containing the C-terminal domain is soluble and does not take part of PNN formation. This fragment strongly regulates the formation of somatostatin-positive inhibitory synapses, which is impaired in mice lacking either the entire *Ncan* gene or just the C-terminal sequence [105].

Collectively these studies indicate that astrocytes might be in a unique position to regulate PNN at multiple scales: from the microenvironment of the synaptic cleft, up to the larger context of the glial scar.

## Microglia: Another Crucial Player Sculpting the ECM

Microglia, the resident immune cells of the central nervous system (CNS), are now known to perform functions far beyond classical immunity [106]. While their roles in pruning synapses during development and stripping them in disease are well-documented [106–108], a growing body of evidence shows they are also integral components of the synaptic microenvironment.

Microglia, the resident immune cells and macrophages of the CNS, are increasingly recognized for functions extending far beyond classical immunity [106]. While their roles in synaptic pruning during development and synaptic stripping in disease are well-studied aspects of their interaction with neurons [106–108], a growing body of evidence shows they are also integral components of the synaptic microenvironment. They are embedded within the complex meshwork of the ECM, which, as mentioned before, together with pre- and postsynaptic terminals and astrocyte processes, forms the tetrapartite synapse [109, 110]. Emerging evidence strongly suggests that microglia actively regulate and remodel both the diffuse perisynaptic ECM and the condensed PNN, adding another layer to their role as sculptors of the neural environment in both health and disease. Microglia possess a variety of instruments enabling them to dynamically interact with and modify the ECM, including PNNs. The two primary mechanisms implicated are direct phagocytosis of matrix components and the secretion of ECM-degrading enzymes.

Direct physical interaction and engulfment of ECM components by microglia is increasingly documented across various contexts. Following peripheral nerve injury (spared nerve injury, SNI model), microglia in the spinal cord dorsal horn become activated and contain WFA immunoreactivity within their lysosomes [111]. Similarly, in mouse models of Amyotrophic Lateral Sclerosis (ALS), activated microglia and astrocytes in the ventral horn show increased engulfment of the core PNN protein aggrecan, coinciding with the timing of PNN breakdown around vulnerable motor neurons [112]. In Alzheimer’s disease (AD) models (5xFAD mice) and human AD brains, microglia associated with amyloid-beta (Aβ) plaques (PAMs) contain intracellular WFA + material and inclusions of PNN components [113]. Finally, loss of PNN coincided with early microglial activation and with a reduction in synaptic plasticity in the hippocampus in a ME7 murine prion disease [114].

While disease states clearly show engulfment, microglia seem to interact with PNNs under baseline conditions. Recent work highlights a fundamental role for microglia cells in clearing perisynaptic ECM (including CSPGs like aggrecan and brevican) via phagocytosis in the healthy adult hippocampus, a process regulated by the neuron-derived cytokine IL-33, which is modulated by experience. Loss of IL-33 or its microglial receptor IL1RL1 impaired ECM engulfment and led to ECM accumulation around synapses associated with impairment in memory consolidation in mice [115]. This suggests microglia perform homeostatic surveillance and a certain extent of ECM clearance, including potentially PNN components, even during health conditions.

Importantly, experimental manipulations are able to activate microglia phagocytic capacity. Repeated anesthetic ketamine exposure, which induces PNN loss, leads to microglia closely interacting with PV + neurons and containing WFA + fragments. Live ex vivo imaging of primary somatosensory cortex coronal slices confirms that microglia cells actively survey WFA-labeled structures and accumulate WFA + material internally over time following ketamine treatment [116]. Although correlative, activation of microglia proliferation by LPS or valproic acid treatment reduces the density of PNNs in both mouse hippocampal CA1 and the visual cortex [117].

Remarkably, postnatal development microglia phagocytic activity of synapses and PNNs in the mediobasal hypothalamus have been implicated in systemic glucose tolerance. In mice, transiently depleting microglia before weaning (postnatal day [P]6–16) but not afterward (P21–31) induces glucose intolerance in adulthood due to impaired insulin responsiveness, which is linked to PNN overabundance and reduced autonomic synaptic connectivity between hypothalamic glucoregulatory neurons and the pancreatic beta cell compartment. The authors conclude that neonatal hypothalamic microglia help program the ability of the CNS to properly potentiate glucose-responsive pancreatic insulin secretion in adulthood by determining the autonomic connectivity of hypothalamic neurons, including those involved in glucose sensing, with the pancreatic beta cell compartment [118].

The signals triggering PNN engulfment are still being elucidated. While classical “eat-me” markers like phosphatidylserine (PS) mediate microglial pruning of synapses, and complement factors (C1q, C3) are involved [119], their specific role in PNN phagocytosis is less clear. The CX3CL1-CX3CR1 neuron-microglia communication axis, important for pruning, does not appear essential for SNI-induced PNN degradation in the spinal cord [111] nor for PNN changes in ALS models [112]. However, the IL-33/IL1RL1 pathway clearly drives ECM engulfment in the hippocampus [115]. It is plausible that damaged or altered PNNs expose signals that may mark them for microglial clearance. A possibility could be related to the sulfation pattern of CSPGs which is sensitive to changes in the microenvironment (e.g. metabolic signals [4, 79]), regulates distinct properties of the matrix [120–122] and may be read as a code for degradation by microglia cells.

Further evidence of the link between ECM remodelling and microglia is that microglia secrete various proteases capable of degrading ECM components, offering an alternative or complementary mechanism to direct phagocytosis. For instance, MMP9 expression is upregulated in microglia (and astrocytes) in the ventral horn of ALS mouse model, coinciding with PNN breakdown. Interestingly, MMP9 inhibition or genetic ablation is protective in ALS models [112]. ADAMTS family members, such as ADAMTS4, are also expressed by microglia and can cleave aggrecan and brevican [123, 124]. However, there is no direct evidence of ADAMTS involvement in microglia-driven reshaping of PNNs and ECM.

We could speculate that microglia dependent enzymatic degradation and phagocytosis work in concert. Proteases released by microglia could ‘prime’ the PNN by cleaving core proteins or GAG chains, creating fragments or altering the structure to make it more amenable to subsequent engulfment.

A striking feature emerging from the above-mentioned studies is that microglia could interact with PNNs and ECM both in health and disease state, suggesting that PNN degradation could be related to homeostatic function as well as to pathological conditions. Thus, the interaction between microglia and PNNs may be tightly regulated with significant functional consequences. Colony stimulating factor 1 receptor (CSF1R) signaling is critical, as both genetic haploinsufficiency as observed in the *Csf1r+/-* model of adult-onset leukoencephalopathy with axonal spheroids and pigmented glia (ALSP), and pharmacological inhibition impact microglial homeostasis and consequently PNN integrity. Loss of one *Csf1r* allele leads to microglial dyshomeostasis (reduced purinergic receptor P2Y G-protein coupled 12, P2RY12) and PNN loss, which can be rescued by eliminating the microglia altogether or, paradoxically, by *further* low-grade CSF1R inhibition through PLX5622. This work suggests that mitigating microglia dyshomeostasis through pharmacological modulation is beneficial to improve the phenotype of ALSP mice [125].

The switch in microglial function from homeostatic maintenance to active PNN degradation may be triggered upon encountering pathological stimuli or inflammatory cues. The precise triggers for this switch likely vary depending on the specific disease context and brain region. It could involve inflammatory signals (e.g. TNFalpha), neuronal stress signals (e.g. CX3CL1, or other chemokines), or direct interaction with pathological proteins like Aβ. Understanding the signals that govern the switch between homeostatic maintenance and pathological degradation of PNNs and ECM in general by microglia holds significant therapeutic potential for a wide range of neurological and psychiatric disorders. Modulating microglial activity, perhaps via CSF1R signaling or through P2RY12, to modulate PNN loss or promote appropriate remodeling could represent a novel strategy to preserve neuronal function, promote synaptic plasticity and combat disease progression.

## Future Directions

In this review, we highlight that PNNs—complex extracellular structures involved in regulating multiple plasticity processes during development and adulthood—require tight regulation to ensure proper circuit maturation, physiological brain function, and responses to pathological states. Increasing evidence shows that PNNs are modulated by signaling molecules, neuronal activity, interactions with astrocytes and microglia, and—more recently—by hormones and metabolic factors. We can speculate that PNNs act as an integratory hub shaping neural plasticity in relation to both brain-intrinsic and peripheral cues. However, further research is needed to elucidate how these regulatory mechanisms cooperate and to identify the central nodes in this network of interactors.

We discussed how astrocytes are involved in the production of ECM molecules and how, together with microglia, control PNNs especially in a pathological context. However, the cooperative dynamics among these cellular players in orchestrating PNN regulation remain poorly understood.

Overall, PNN deposition is not solely determined by the cells enwrapped by PNNs themselves, but depends on multiple cell types and, in some cases, on signals originating from distant brain regions or even peripheral tissues. For example, in the brain, many—but not all—PV interneurons are surrounded by PNNs [38], suggesting that PNN expression is driven by mechanisms not shared across the entire cell type. Nevertheless, the molecular signals or cellular machinery that enable specific neurons to be ensheathed by PNNs remain largely unknown.

Another major challenge in studying PNN regulation stems from the complex biology of CSPGs. For instance, the sulfation pattern of CSPG sugar chains—a critical factor influencing PNN properties during development— is studied as a molecular code modulating plasticity, while other sugar modifications and their impact on PNN physiology and plasticity have not been characterized. Recent methodological advances offer promising solutions. For example, spatial omics approaches, including spatial transcriptomics [126] and proteomics [127, 128], can help characterize the molecular fingerprint of PNN-ensheated cells in different brain regions as well as the biochemical pathways responsible for their aggregation. Additionally, expansion microscopy [129, 130] could enable high-resolution imaging of ECM structures and glycan modifications within brain tissue, offering unprecedented insights into PNN architecture and molecular composition. We anticipate that these tools will significantly deepen our understanding of the molecular regulation and functional diversity of PNNs.

The complexity of PNN regulation is further compounded by regional differences in PNN composition and dynamics across brain areas. It is conceivable that region-specific connectivity patterns might cause rearrangements of PNN both in development or in adulthood, perhaps driving activity of the local circuits towards a more or less plastic configuration.

In addition to this, while attention has been focused on condensed PNNs, the regulation of the diffuse ECM remains poorly understood, as its role in neural plasticity control.

Technical advancements are already promising, particularly in the field of optical methods for the visualization of PNNs in vivo, either with fluorescent labeling of WFA or through transgenic models [131, 132], which could provide valuable means to test longitudinally the effects of specific modulatory elements on PNN biology. Recent work also showed the possibility of studying the PNN interactome by performing precipitation with WFA and mass spectroscopy analysis [86]. Nevertheless, the development of new tools for the functional and molecular characterization of PNNs will be needed to further clarify their tight control of neural plasticity in health and disease.

## Data Availability

No datasets were generated or analysed during the current study.
